# Recent Progress in TRPM8 Modulation: An Update

**DOI:** 10.3390/ijms20112618

**Published:** 2019-05-28

**Authors:** Rosario González-Muñiz, M. Angeles Bonache, Cristina Martín-Escura, Isabel Gómez-Monterrey

**Affiliations:** 1 Instituto de Química Médica, IQM-CSIC. Juan de la Cierva 3, 28006 Madrid, Spain; angelesbonache@hotmail.com (M.A.B.); cristinamartinescura@gmail.com (C.M.-E.); 2 Dipartimento di Farmacia, Università “Federico II” de Napoli, Via D. Montesano 49, 80131 Naples, Italy

**Keywords:** TRPM8, agonists, antagonists, structure

## Abstract

The transient receptor potential melastatin subtype 8 (TRPM8) is a nonselective, multimodal ion channel, activated by low temperatures (<28 °C), pressure, and cooling compounds (menthol, icilin). Experimental evidences indicated a role of TRPM8 in cold thermal transduction, different life-threatening tumors, and other pathologies, including migraine, urinary tract dysfunction, dry eye disease, and obesity. Hence, the modulation of the TRPM8 channel could be essential in order to understand its implications in these pathologies and for therapeutic intervention. This short review will cover recent progress on the TRPM8 agonists and antagonists, describing newly reported chemotypes, and their application in the pharmacological characterization of TRPM8 in health and disease. The recently described structures of the TRPM8 channel alone or complexed with known agonists and PIP_2_ are also discussed.

## 1. Introduction

The cold- and menthol-sensitive transient receptor potential melastatin 8 (TRPM8) receptor is a nonselective cation channel, with a certain preference for Ca^2+^ permeation [1,2]. It shows multimodal gating which is activated by cold (<28 °C), membrane depolarization, different cooling compounds such as menthol (**1**) and icilin, among others, and changes in extracellular osmolality [1,2]. While phosphoinositide PIP_2_ is a key regulator of channel gating [3,4], testosterone [5], artemin [6,7], and Pirt (phosphoinositide interacting regulator of TRP) protein [8] have been proposed as endogenous ligands of TRPM8. These channels are highly expressed in peripheral sensory neurons (Aδand C fiber afferents), and also on deep visceral afferents in prostate, bronchopulmonary tissue, bladder, and the urogenital tract. 

It is known that TRPM8 receptors are implicated in cold allodynia after inflammation or nerve injury, and different modulators have been studied as potential treatments for different pain conditions [9]. In addition, some studies strongly suggested a correlation between specific Single Nucleotide Polymorphisms SNPs in the TRPM8 encoding gene and migraine processes, thus positioning TRPM8 as a potential novel target for this disabling condition [9,10].

Dry eye disease (DED), a growing problem today, due to increased pollution and the use of electronic screens, among other factors, produces persistent pain on the ocular surface [11]. Although the mechanisms underlying this symptom remain unclear, the implication of TRP channels is well recognized. In fact, TRPV1 channels have been described as having a significant role in mediating enhanced nocifensive behaviors, while the use of TRPM8 agonists could relief the associated issues through the increased production of tears [12]. 

Numerous experimental results link the expression of the TRPM8 channel with cell migration and tumor progression [13,14]. The upregulation of this channel in different tumors, such as prostate, pancreas, colon, breast, lung, and skin is well documented, and in most cases the expression correlates with tumor aggressiveness [15]. However, while this seems true for initial phases, it has also been proposed that TRPM8 could have a protective role in advanced metastatic stages. To solve these discrepancies, there is a need of efficient TRPM8 modulators, both agonists and antagonists, and of TRPM8 diagnostic probes to shed light on the real role of these channels in cancer malignancies.

In addition to the above mentioned pathologic processes, TRPM8 channels have also been implicated in irritable bowel syndrome (SNP increased risks) [16], oropharyngeal dysphagia (OD) [17], and chronic cough [18], while the downregulation of TRPM8 by angiotensin II may be involved in hypertension [19]. Therefore, the TRPM8 receptor could be considered a valuable therapeutic target to develop new active pharmacological treatments for all these pathologies.

Due to these experimental results on the participation of the TRPM8 channels in the above-mentioned pathologies, it is not surprising that numerous research groups, both academic and pharmaceutical companies, have become interested in the pharmacological modulation of these receptors. In recent years, several review articles have been published that have covered either agonists, antagonists, or both [9,20,21,22,23]. This compendium is a follow-up of our previous revision [24], and it is aimed to update the knowledge in the last few years, and to summarize the structural knowledge that have arisen recently, thanks to the advances in electron cryo-microscopy.

## 2. TRPM8 Agonists

**Figure 1:**
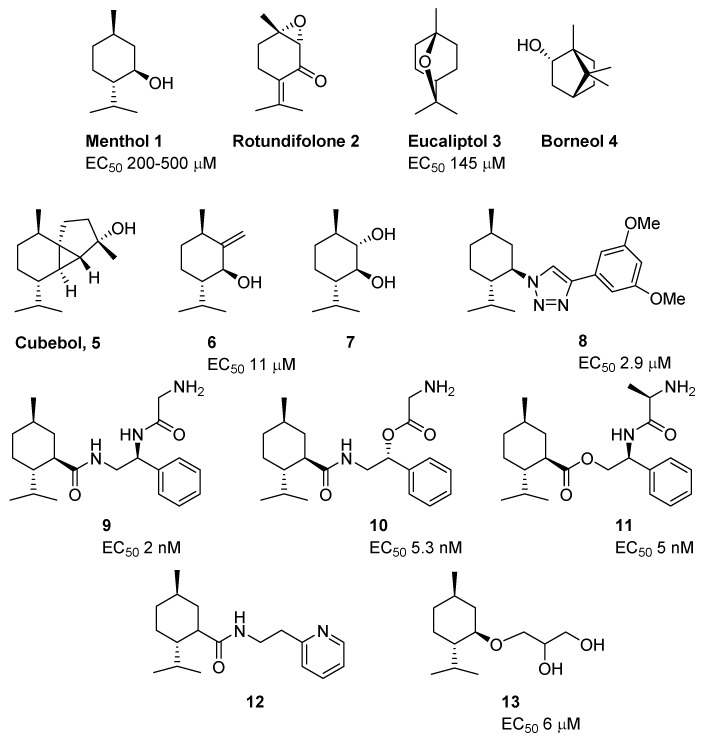
Natural products and recently described menthol-derived TRPM8 agonists.

Natural products are considered a powerful tool for the identification of functional roles of biomolecules, in our case the TRPM8 channels, as well as an essential source for the development of new drug discovery programs [25]. This section compiles natural and synthetic chemotypes identified in recent times as TRPM8 agonists, along with previous agonists that have recently been used to explore the pharmacological role of this channel, in addition to menthol (**1**, Figure 1).

An example is rotundifolone (**2**, Figure 1), a natural monoterpene found in several species of the genus *Mentha L.*, with interesting antinociceptive activity [26]. Recently, Silva et al. demonstrated that this compound activates the TRPM8 channel more selectively than menthol, and causes Ca^2+^ influx across the plasma membrane at a concentration of 0.3–3 mM [27]. Rotundifolone produces vasorelaxation in rat mesenteric artery and improves this response when it is induced by low temperature. The pretreatment with TRPM8 channel blockers, such as capsazepine or 4-(3-Chloro-2-pyridinyl)-N-[4-(1,1-dimethylethyl)phenyl]-1-piperazinecarboxamide (BCTC), significantly attenuated these effects. 

Two different studies confirmed that eucalyptol (1,8-cineol, **3**) is an effective tool to investigate the biology of TRP channels [28,29]. Thus, Urata et al. established the relationship between thermoregulation and TRPM8 expression in the afferent vagal nerve [28]. In mice, intragastric and intravenous administration of 1,8-cineol (100 and 10 mg/kg, respectively) increased the temperature of the intrascapular brown adipose tissue and colon, whereas, previous treatment with a TRPM8 antagonist, M8-B, inhibited these responses. This change in body temperature was also attenuated in vagotomized mice, suggesting that the vagal nerve also participates in thermoregulation, other than the cutaneous sensory nerves. On the other hand, a second study demonstrated the involvement of TRPM8 channels in the anti-inflammatory effects induced by eucalyptol, both in lungs of mice exposed to lipopolysaccharide (LPS) and in a Complete Freund’s Adjuvant (CFA)-induced model of inflammatory pain [29]. At concentrations of 200 and 300 mg/kg, respectively, eucalyptol attenuated the inflammation and showed strong analgesic effects. These anti-inflammatory and analgesic effects were completely absent in TRPM8 deficient mice. In addition, the authors highlight different sensitivities of TRPM8 channel species orthologues (human, mouse, and rat) to eucalyptol, measured by Ca^2+^ fluorimetry in HEK293T cells, with EC_50_ values of 145.6, 924.5, and 1210.0 μM, respectively.

A recent patent claimed a series of pharmaceutical forms, which include mainly menthol (**1**) and eucalyptol (**3**), together with other anti-inflammatory natural products, as effective treatments for inflammation and different painful processes [30]. The inventors rationalized that the activation of TRPM8, the inactivation of transient receptor potential cation channel, subfamily A, member 1 (TRPA1) without activating the transient receptor potential cation channel, subfamily V, member 1 (TRPV1) ion channels, and the inhibition of the enzyme cyclooxygenase 2 (COX-2) effectively blocked pain signaling to the brain from the skin, muscles, and joints. 

Borneol (**4**, Figure 1), another terpene derivative, activates TRPM8 channels in a temperature- and dose-dependent manner in the range of 10 μM to 2 mM, without affecting cell viability [31]. In vivo, 100µM Borneol significantly increased tear production in guinea pigs without evoking nociceptive responses at 25 °C, however, it was not effective at 35 °C. As expected, TRPM8 channel blockers, N-(3-aminopropyl)-2-[(3-methylphenyl)methoxy]-N-(2-thienylmethyl)-benzamide hydrochloride (AMTB) and BCTC, abolished this effect [31]. 

A series of exocyclic olefin analogues of menthol have also been described [32]. They were identified when performing the total synthesis of cubebol (**5**, Figure 1), a natural sesquiterpene patented as a cooling agent by Firmenich SA [33]. The allylic alcohol (1S,3R,6S)-6-isopropyl-3-methyl-2-methylenecyclohexanol (**6**, Figure 1), intermediate of the cubebol synthetic route, showed TRPM8 activation with an EC_50_ value of 11 ± 1 μM when tested in both the Ca^2+^ assays and electrophysiology. Structural modification of this compound and a SAR study confirmed **6** (Figure 1) as the most potent agonist in this series, and identified both an antagonist (see next section compound **51**) and the trans diol derivative **7** (Figure 1) as an allosteric modulator that minimized the menthol-induced channel desensitization. 

Ferrer-Montiel et al. developed a series of triazole-based menthol derivatives as potent TRPM8 agonists, and they claimed that they could be particularly useful in the prevention and treatment of dry eye syndrome (DES), vaginal dryness, and burning mouth syndrome [34]. In particular, compound 4-(3,5-dimethoxyphenyl)-1-((1*R*,2*S*,5*R*)-2-isopropyl-5-methylcyclohexyl)-1H-1,2,3-triazole (**8**, Figure 1) shows high specificity in activating the TRPM8 channels (EC_50_ = 2.91 μM) in relation to other thermoTRP channels such as TRPV1 and TRPA1. In this series, the presence of bulky groups at triazole position 4, such as biphenyl or phenethyl, afforded compounds that maintain the activity (EC_50_ = 3.47 and 3.84 μM, respectively), while groups such as pyridine or dimethoxy phenyl methanone induced a weakly decrease in channel activation, although they still maintained EC_50_ values in the micromolar range (EC_50_ = 6.23 and 9.84 μM, respectively). In an in vivo assay, the treatment with 10 µM of **8** (Figure 1) induced a significant increase in the lacrimation rate in rat, similar to that produced by 200 µM menthol.

The Procter and Gamble Company expanded its menthol-based carboxamide and ester series of derivatives with two new patents [35,36]. One of the most potent compounds of the carboxamide series, with EC_50_ < 2nM, is (1*R*,2*S*,5*R*)-N-((*S*)-2-((*S*)-2-aminopropanamido)-2-phenylethyl)-2-isopropyl-5-methylcyclohexanecarboxamide (**9**, Figure 1). In this series, substitutions of glycine by *L*-Ala or of the NH at position 2 by an oxygen (compound **10**, Figure 1) are well accepted, since the corresponding derivatives maintained the activity in the nanomolar range (EC_50_ = 5.0 or 5.3 nM, respectively). In contrast, the incorporation of a *D*-Ala residue led to a 12-fold less powerful compound (EC_50_ = 66.0 nM). In addition, modifications on the aryl moiety, by introducing benzyl, cyclohexyl, or 2-chlorophenyl groups, resulted in significantly decreased activity of micromolar values. In the ester series, the nature of the amino acid residue is still more important. The most potent compound **11** (Figure 1), containing the amino acid *D*-Ala, induced TRPM8 activation at nanomolar concentrations (EC_50_ = 8.1 nM), while Gly derivative dramatically decrease its activity (EC_50_ = 0.34 µM). Here, the configurational change in the amino acid residue (*D*-Ala to *L*-Ala) made the corresponding derivative eight times less potent (EC_50_ = 64 nM).

Several clinical studies are currently evaluating the efficacy of TRPM 8 agonists as therapeutic agents, alone or in combination with other drugs. Thus, the effects of a lotion that includes two menthol-based TRPM8 agonists, (1R,2S,5R)-N-(2-(2-pyridinyl)ethyl)-2-isopropyl-5-methylcyclohexancarbox-amide (**12**, Figure 1) and menthoxypropanediol (MPO) (**13**, Figure 1), prepared a few years ago [37], has been evaluated in patients suffering from chronic pruritus due to dry skin (ClinicalTrials.gov: NCT00669708) [38]. This lotion showed stronger activation of the TRPM8 channels than menthol and ameliorates severe pruritus, whereby representing a potential treatment for this burdensome symptom. More recently, Misery et al. evaluated the anti-itching effects of a cream containing only MPO (**13**, Figure 1) in patients with atopic dermatitis (AD) in real-life settings. In this clinical study, MPO cream was effective against itch, with a rapid effect and more than 90% of patients satisfied after 15 min or longer. Neither of these two studies indicated the concentrations of active principle that was used [39]. In cell models, Roggenkamp et al. identified compound **13** (Figure 1) as a potent inhibitor of the nuclear factor kappa-light-chain-enhancer of activated B cells (NFκB)-mediated signal transduction pathway which promotes inflammatory processes [40]. Compound **13** (Figure 1) inhibited neuritis outgrowth in dermal fibroblast treated with histamine, as well as the germination of nerve fibers in innervated skin models, including atopic fibroblasts and normal keratinocytes, by reducing neuronal growth factor (NGF) expression. Given that elevated NGF levels and increased cutaneous nerve fiber density are associated with itch [41], these results point to the conclusion that MPO-induced anti-inflammatory effect is based not only on the activation of TRPM8 cold-sensing nerve fibers [42], but also on modified crosstalk between nerve endings and skin cells. These authors suggest that an alternative mechanism to explain the modulation of the anti-inflammatory activity of this cooling agent could include a reduction of inflammation-induced neuronal G protein-coupled receptors (GPCRs) release.

One of the more interesting applications for TRPM8 modulators has been described by Melior Pharmaceuticals I, Inc [43]. This company claimed different compositions containing a Lyn kinase activator, with structures of 5-phenoxypyrimidinone or 5-phenoxypyrimidinediones, and well-known TRPM8 agonists [24], such as menthol, icilin, N-[6-[4-[[[[4-[(4-Methyl-1-piperazinyl)methyl]-3-(trifluoromethyl)phenyl]-amino]carbonyl]amino]phenoxy]-4-pyrimidinyl]-cyclopropanecarboxamide (WS3), andN,2,3-trimethyl-e-isopropylbutanamide (WS23). Potential uses for these combinations include reduction of blood glucose levels, weight gain, or fat depot levels and treatments for metabolic syndrome, obesity, prediabetes, and type II diabetes. Other applications have been claimed, such as treating hypercholesterolemia, hypertension, coronary heart disease, diabetic neuropathy and retinopathy, erectile dysfunction, kidney disease, and pancreatitis, among others. 

**Figure 2:**
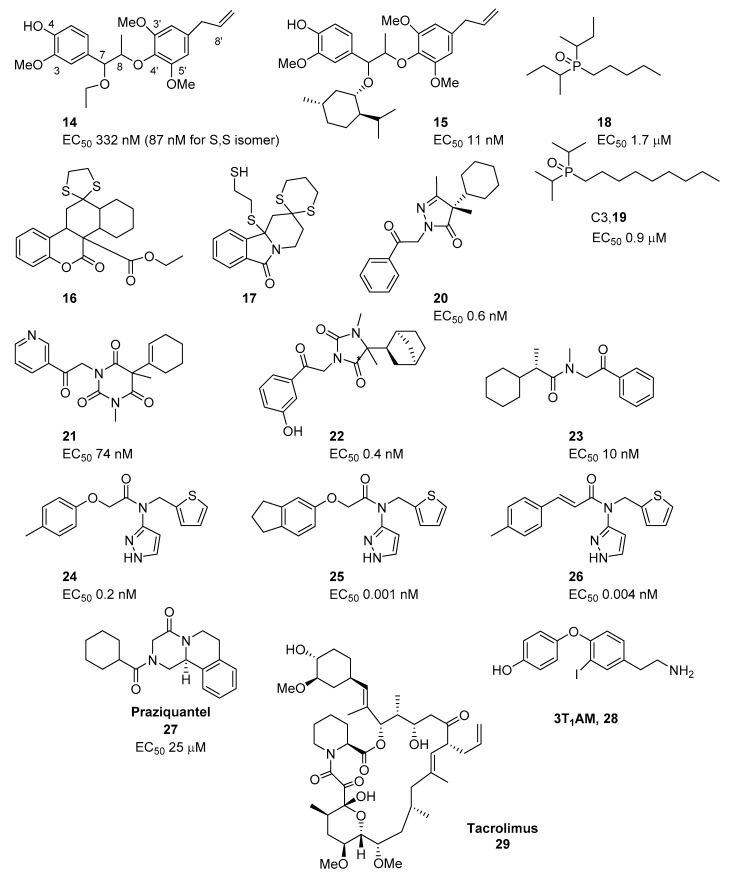
Recently described non-menthol chemotypes acting as TRPM8 agonists.

Several non-menthol derivatives have also been described. Therefore, Shirai et al. reported the isolation and identification of a new neolignan from nutmeg (*Myristica fragrans* Houtt), the *erythro*- and *threo*-Δ8,7-ethoxy-4-hydroxy-3,3′,5′-trimethoxy-8-O-4′neolignan (**14**, Figure 2), as well as its agonist activity on the TRPM8 channels [44]. The diastereoisomer mixture activated the channel at EC_50_ of 332 nM, while the 7S, 8S diasteroisomer was 4-fold more potent with an EC_50_ = 87 nM. Structure activity relationships revealed that the 4-hydroxy group is essential, while longer or bulkier alkoxy groups at C7 are also important for activity. However, the 7-O-*d*-menthoxy derivative (**15**, Figure 2) is the most potent agonist in the series (EC_50_ = 11 nM). The combination of **15** (Figure 2) and l-(−)-menthol has an additive effect, suggesting that neolignan compounds interact with TRPM8 at different sites from that of menthol.

BASF extended its previous patents [45,46], and therefore increased the TRPM8 agonist chemotypes useful for application in the pharmaceutical field (tumor treatment, and bladder weakness, among others) [47]. A series of decahydronaphtho[1,2-*c*]chromene-based compounds, such as **16** (Figure 2), or of dihydropyrido[2,1*a*]isoindolone derivatives, represented by **17** (Figure 2), were evaluated using hTRPM8 expressed in human embryonic kidney (HEK) 293 cells (Figure 2). According to the inventors, some of these derivatives exhibited better activity than menthol and were as effective as icilin, however, potency values were not disclosed.

In 2015, Wei patented a series of di-isopropylphosphinoyl alkane (DIPA) derivatives as topical agents for the treatment of skin and ocular discomfort [48]. In a more recent patent, the same author claimed several compositions containing these di-alkylphosphinoyl alkanes for treatment of lower gastrointestinal tract disorders (LGITD) [49]. The author demonstrated for the first time that hTRPM8 is responsive to 1-di(*sec*-butyl)phosphinoyl pentane (DAPA-5, **18**, Figure 2) in the colon and, in general, that these phosphine oxide derivatives can be used to treat LGIT dysfunction. Contrarily to menthol, these compounds exerted a potent TRPM8-dependent inhibitory effect on the spontaneous mechanical activity of human circular smooth muscles cells, with a mean inhibitory concentration of 8 µg/mL or EC_50_ = 34 µM. In the in vivo experiments, the compound with the best biological profile was the DIPA derivatives with a linear chain of 9 carbons (C3, **19**, Figure 2), followed by the DAPA analogues with aliphatic chains of 5–8 carbon atoms. This water-soluble cryosim-3 (C3) (**19**, Figure 2) was used for the topical treatment of patients with mild forms of dry eye disease (DED) (TR: ISRCTN24802609 and ISRCTN13359367) [50]. Treatment with a 2 mg/mL solution of **19** (Figure 2) in water for two weeks, four times a day, resulted in an increase of basal tear secretion in these patients, which showed improvement of symptoms at one and two weeks, without reporting irritation or pain.

Researchers at Senomyx, Inc. developed five series of new potent TRPM8 agonists from the following scaffolds: pyrazol-5-one, pyrimidine-2,4,6-trione, imidazolidine-2,4-dione, 2-phenyl propanamide, and phenoxyacetylamide (Figure 2) [51,52]. In cell line expressing hTRPM8, representative examples of these series, such as compounds **20**, **21**, **22**, **23**, and **24** (Figure 2) showed EC_50_ values of 0.6, 74, 0.4, 10, and 0.2 nM, respectively. The chirality of **20** and **22** (Figure 2) strongly affected the activity of these compounds. Thus, compound **20** (Figure 2) with *S* configuration at C-4 is 145 times more potent than its *R* enantiomer (EC_50_ = 87 nM), while the most potent epimer at C-5 of **22** (Figure 2) (exact configuration not defined) has an activity 250 times higher than the R,S mixture [52]. Structure-activity relationship (SAR) studies on the series of phenoxyacetamide derivatives identified both the 3-pyrazoyl and the thiophene rings as essential moieties for the activity [53]. Substituents at para position of the aromatic ring, in particular the 4-methyl group, are optimal for high levels of activity (**24**, EC_50_ = 0.2 nM, Figure 2). Bridging the 3 and 4 positions of the aromatic ring to form a five-membered carbocyclic ring resulted in a further improvement of activity (**25**, EC_50_ = 0.001nM, Figure 2), while switching of the –OCH_2_– linking group between the phenyl ring and the amide carbonyl to a double bond afforded new acrylamide derivatives, as **26** (EC_50_ = 0.004nM, Figure 2), although their pharmacokinetic properties are not optimal yet. Some related analogues, but showing micromolar potency, have been patented by BASF [54].

Drug repositioning, or the search for new uses for old drugs, is a popular strategy today due to its high efficiency, and low cost and risk. Following this strategy, in 2017 Babes et al. reported the anthelminthic drug praziquantel (PZQ, **27**, Figure 2) as a selective micromolar agonist of the TRPM8 channels [55]. PZQ, such as menthol, activated wild-type cells but not the Y745H mutant hTRPM8 expressing cells. However, this compound inhibited TRPM8 when activated by the full agonist menthol, an effect consistent with a partial agonist/antagonist activity. PZQ only slightly activated TRPV1 at the highest concentration tested (>100 μM), while it had no effect on TRPA1. In addition, PZQ evoked calcium transients in a subpopulation of dorsal root ganglion (DRG) neurons, which were also sensitive to the selective TRPM8 agonist WS-12. The TRPM8 antagonist AMTB, strongly inhibited this effect. However, the authors did not provide evidence for direct PZQ binding to TRPM8, and they suggested that these results could also be compatible with a model in which the TRPM8 channel is a downstream effector of another primary binding target of PZQ. In a later work, Gunaratne et al. reported that PZQ acts as a partial agonist of hTRPM8 in the micromolar range (EC_50_ = 19 ± 5 μM), is also a weak TRPA1 agonist, while it is ineffective on TRPV1 [56]. In addition, PZQ induced a vasodilator effect in mesenteric vessels, an effect associated with TRPM8 activation [57]. The TRPM8 activation and the relaxing effect in mesenteric arteries are both mediated exclusively by the (S)-PZQ enantiomer. However, the extent of relaxation was similar in WT and TRPM8 KO tissues, suggesting that the relaxation observed with the TRPM8 agonists and (*S*)-PZQ was probably caused by its action on other targets.

In the last decade a significant number of studies identified a metabolite of the thyroid hormone, the 3-iodothyronamine (3-T_1_AM, **28**, Figure 2), as an interesting TRPM8 agonist [58]. 3-T_1_AM directly activated TRPM8 in rat thyrocyte (PCCL3 cells) [59], human conjunctival epithelial cells (HCjEC) [60], and a murine hypothalamic cell line [61]. In all cases, 3-T1AM induced Ca^2+^ responsed similar to that of the specific TRPM8 agonist menthol. In ocular cells, 3-T1AM induced both Ca^2+^ mobilization and increases in whole-cell currents [60]. These stimulatory effects could be specifically reduced in the presence of AMTB, a TRPM8 blocker. On the other hand, an inhibition of TRPV1-induced Ca^2+^ influxes by 3-T_1_AM-based TRPM8 activation through a negative feedback was observed in human corneal epithelial cells [62], and cancer cells, such as TRPM8 transfected U2osB2 osteosarcoma [63], WERI-Rb1 retinoblastoma, and human uveal melanoma (UM 92-1) [64]. The effects observed in these cells showed a complex co-regulation between TRPs and some members of the GPCR receptor family, which are also targets of this metabolite [65]. This close crosstalk of TRPs and GPCRs offers new opportunities for therapeutic intervention in pathophysiological conditions involving these systems, and also increases the interest for the thyroid hormone metabolites as lead compounds in the search of more potent modulators of the TRPM8 channels.

The group of Viana recently described that the immunosuppressant Tacrolimus (**29**, Figure 2) activates the TRPM8 channels in different species, increasing intracellular calcium through cold-sensitive neurons, a response that was significantly reduced in TRPM8 *knockout (KO)* mice or by application of selective antagonists [66]. Although this macrocyclic compound caused blinking and cold-evoked behaviors, its activity on menthol and icilin-insensitive mutants suggested a binding site different to that of the small-molecule natural products.

Recently, it was demonstrated that oxidative stress and ADP-ribose induced intracellular Ca^2+^ responses in certain tumor cell lines (prostate and kidney), and increased apoptosis, annexin V, intracellular reactive oxygen species (ROS), and caspase 3 and 9 values [67].

It is interesting to note that Voets et al. proposed classification of the TRPM8 agonists into two groups, type I (menthol-like) and type II (allyl isothiocyanate, AITC-like), and provided different kinetic models for both types (type I stabilizes the open channel while type II destabilizes the closed channel) [68]. This finding should be taken into account for future understanding of differential actions by different TRPM8 agonists.

## 3. TRPM8 Antagonists

**Figure 3:**
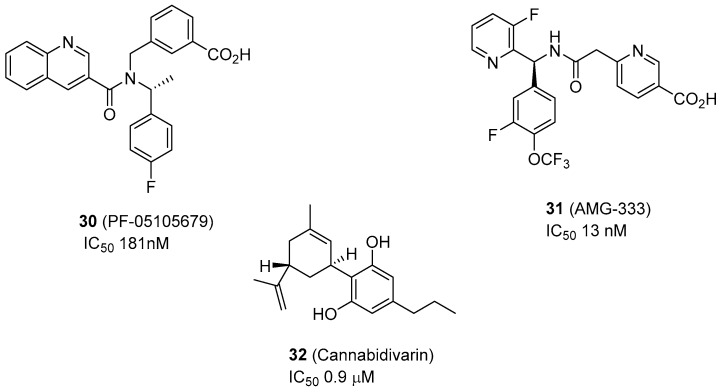
Selective TRPM8 antagonists that have reached clinical trials.

In the recent decade, numerous TRPM8 antagonists have been reported by academic groups, as well as pharmaceutical and biotech companies, as potential drugs for neuropathic pain, inflammation, migraine, and cancer [24]. However, most antagonists described in the literature lack selectivity for TRPM8, interacting also with TRPV1 and TRPA1. Only three compounds have reached clinical trials to date, PF-05105679 (**30**, Figure 3) and AMG-333 (**31**, Figure 3) which have not passed phase I studies [69], and Cannabidivarin, (**32**, Figure 3) which is in phase II clinical assays (Figure 3) [69,70]. For this reason, it is necessary to discover new, potent and selective TRPM8 antagonists, and to increase our knowledge about their binding sites on the target protein, which now will be facilitated by the publication of the first TRPM8 structures by electron cryo-microscopy (see Section 4 for a detailed description). In this section we summarize the progress accomplished in the search of the TRPM8 antagonists in the last four to five years.

**Figure 4:**
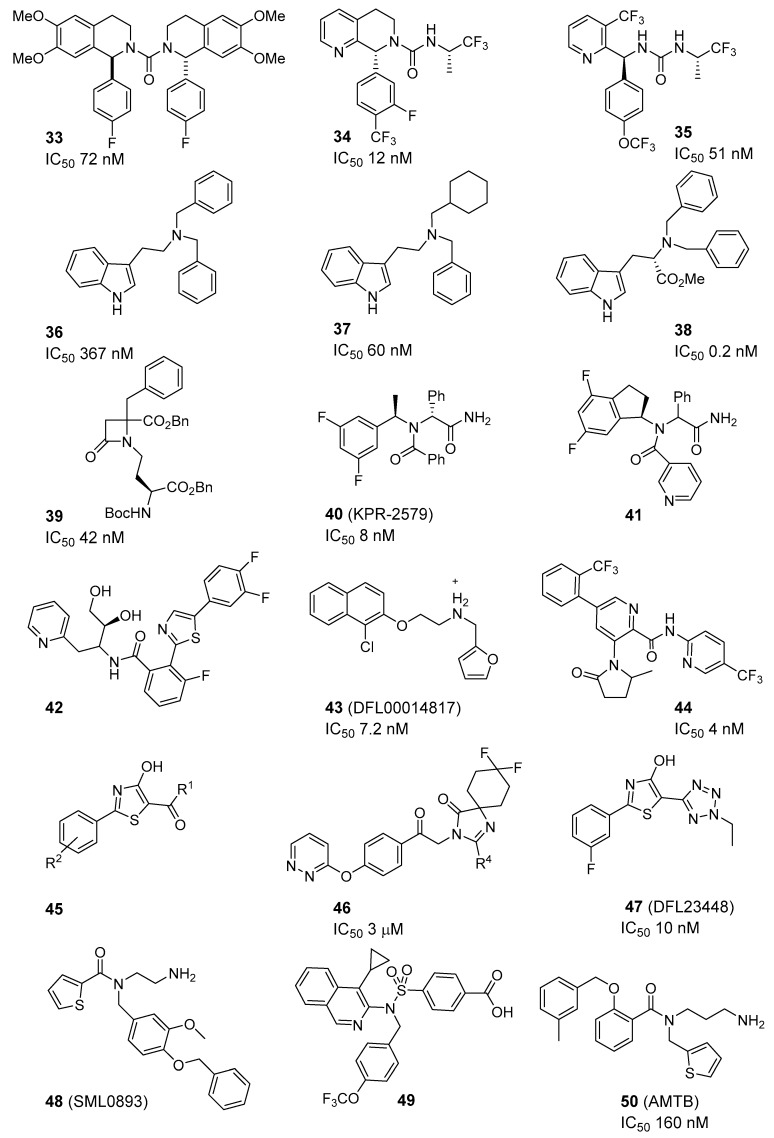
Lately described TRPM8 antagonists.

De Petrocellis’s group recently reported two new families of compounds which are able to antagonize the TRPM8 channels, namely, two series of tetrahydroisoquinoline-derived ureas (symmetric and asymmetric) and another of pyrazino[*1,2-b*]isoquinolin-1-ones (39 new compounds in total), confirming that both the urea function and the tetrahydroisoquinoline ring are important for activity [71]. In general, symmetric bis-tetrahydroisoquinolines showed the best activities, with trans-isomers being more potent than their cis-counterparts, and with a preference for aryl (phenyl) groups at C-1 of the isoquinoleine moiety, as compared with alkyl substituents (n-Pr, Et). The most potent compound, bis (1-*p*-Fluorophenyl-6,7-dimethoxy-1,2,3,4-tetrahydroisoquinoline) (**33**) displayed an IC_50_ value of 72 nM (Figure 4), and the presence of p-halogens in the phenyl group at isoquinoleine C-1 was favorable in general (4F ˃ 4CF_3_ ˃ 4Cl ˃ 4Br˃ 2Cl). Compound **33** (Figure 4) showed good TRPM8/TRPx selectivities (1000:1), antiproliferative activity in a LNCap cell line of prostate cancer (64.3% cellular growth inhibition after three days) and apoptotic activity (enhancement of caspase 3/7) [71]. A related tetrahydronaphthyridine-derived asymmetric urea, (**34**, Figure 4) described by Amgen, is a potent TRPM8 antagonist and demonstrated good efficacy in inhibiting wet-dog shaking and cold-induced arterial blood pressure in the corresponding in vivo rat models [72]. Unfortunately, this compound was not able to mitigate the neuropathic pain in rat models of tactile allodynia and the inflammatory mechanical hypersensitivity [73]. The same company discovered that the linear urea derivatives used as intermediates in the synthesis of tetrahydronaphthyridines, such as **34** (Figure 4), represented a suitable scaffold in the search for new TRPM8 antagonists [74]. In these intermediates, the substitution of the 3-Br group by a 3-CF_3_ moiety improved moderately the blockade activity (IC_50_ = 59 nM versus IC_50_ = 51 nM, **35**, Figure 4), while other groups such as F, allyl, and propynyl were also well tolerated. Then, a library of urea (203 components) and amides (52 derivatives) was prepared from compound **35** (Figure 4). In general, amides were more potent than urea and different carboxylates were selected for the SAR exploration. They observed, for the amide moiety, that 2-pyridone-5-carboxylates and nicotinic acids led to the best compounds in the series, with nanomolar IC_50_ values in blocking hTRPM8, and good in vivo pharmacokinetic (PK) properties. AMG333, (*S*)-6-(((3-Fluoro-4-(trifluoromethoxy)phenyl)(3-fluoropyridin-2-yl)methyl)carbamoyl)nicotinic acid (**31**, Figure 3) was nominated as a clinical candidate for the treatment of migraine (reached phase I human clinical assays, no news since April 2015). Its properties are: hTRPM8 IC_50_ = 13 nM, clogD (pH 7.4) = 0.13, minimal CYP3A4 induction 1µM, rat iv CL(CL_ub_) = 0.32 (11) L/h/Kg, rat %F = 71 [74].

The group of Gómez-Monterrey described a series of tryptamine derivatives with the TRPM8 antagonist activity in the high nanomolar range (patch clamp), exemplified by compound **36** (Figure 4) [75]. Further optimization cycles led first to the discovery of analogue **37** (Figure 4), containing a methyl cyclohexyl moiety instead of a benzyl group, more potent and effective than the prototype **36** (Figure 4) [76]. In this new series, the conformational restriction of the *N-N’*-dibenzyl group into a 2,3-dihydro-1*H*-benzoisoquinoline ring resulted in a significant loss of activity, but the restriction to a β-carboline maintained the blockade potency. More importantly, the replacement of the tryptamine scafold by an L-tryptophan led to the highly potent derivative **38**, (Figure 4) showing sub-nanomolar potency (IC_50_ = 0.2 ± 0.2 nM, Figure 4), and suggesting a key role of the methoxycarbonyl group for the interaction with the TRPM8 channels. Similarly, the four-orders of magnitude lower activity of its D-enantiomer is indicative of a crucial importance of the chiral center. In mice models, pretreatments with compound **38** (Figure 4) reduced oxaliplatin-induced cold allodynia (at 0.1–1 µg s.c.) and icilin-induced wet-dog shakes (at 1–30 mg/Kg s.c.). Molecular modelling studies identified a possible binding site, suggesting that these antagonists could lead to a perturbation in the interaction network established among the TRP domain and the S1–4 transmembrane segments of channel subunits [76].

A library of highly substituted azetidin-2-ones, prepared from amino acid conjugates, behave as the TRPM8 antagonists, by blocking all modalities of channel activation, voltage, menthol, and temperature [77]. After high-throughput screening and SAR studies, it was concluded that preferred substituents on the β-lactam ring are hydrophobic groups (Bn ˃ ^t^Bu) and that short N-alkyl chain (≤3 carbons) are needed. The pharmacological characterization of the prototype β-lactam **39** (Figure 4) showed nanomolar potency in patch-clamp experiments (IC_50_ = 42 nM) and a high selectivity toward TRPV1, TRPA1, Kv1.1, and NaV1.6. Molecular modelling studies on a homology model generated from the CryoEM structure of TRPV1 (the TRPM8 structure was not disclosed at that moment) suggested a negative allosteric modulation of the channel by this family of compounds [77].

Anticholinergic agents are used in the therapy of overactive bladder syndrome (OAB), but these drugs are associated with serious adverse effects. Currently, there are some reports indicating the interest of the TRPM8 antagonists in urological disorders. For example, Kissei Pharmaceutical Co. described novel α-arylglycinamides, as the TRPM8 antagonists, playing important roles in bladder diseases. Thus, the structure-activity relationships of a number of phenylglycine derivatives led to compounds KPR-2579, **40** (IC_50_ = 0.080 ± 0.005 µM), a TRPM8 selective antagonist, which do not show cardiovascular effect at the effective dose, and decrease the number of icilin-induced wet-dog shakes and rhythmic bladder contraction in rats [78]. Ulterior studies confirmed, in pathologies related with hypersensitive bladder disorders in rats, that compound **40** (Figure 4) inhibited acetic acid-induced bladder afferent hyperactivity without affecting body temperature, and therefore is considered a good candidate for future development [79]. In addition, some related phenylglycine amides, having a *N*-inden-1-yl ring and *N*-arylacyl moieties (selected from aryl and 5- and 6-membered heterocyclic rings) have been recorded in a patent by the same company [80]. Within them, compound **41** showed potent inhibitory effect on icilin-induced wet-dog shaking in rats and was claimed useful for the treatment of LUTS (lower urinary tract symptoms) (Figure 4). Kissei Pharmaceutical has also described pyrazole and 2-(phenylthiazolyl)benzamide derivatives as TRPM8 antagonists useful for the treatment of conditions related to the hiperexcitability of afferent nerves, particularly, in OAB and LUTS. For example, compound **42** (Figure 4) at 1 mg/kg/mL intravenous exhibited a 162% elongation of the micturition interval in female rats [81,82].

Using a combination of ligand- and structure-based virtual screening strategy, Beccari et al. identified a new family of naphthyl derivatives behaving as selective and potent TRPM8 antagonists. They screened virtually a library of 124.107 highly diverse compounds, and identify 11.725 promising active molecules that were pharmacologically characterized (Ca^2+^-sensitive florescent dye assay). From the family of active naphtyl derivatives, they selected compound **43** (Figure 4) displaying nanomolar potency (IC_50_ = 7.23 nM); selectivity towards TRPA1, TRPV1, and TRPV3; good pK properties; and in vivo efficacy in an overactive bladder rat model [83]. The authors claimed that this family of compounds could be developed as clinical candidates in the treatment of pain hypersensitivity that was associated with bladder disorders.

Due to the interest of TRPM8 antagonists, other pharmaceutical companies also focused their efforts on the synthesis of new chemotypes. Thus, Mitsubishi Tanabe Pharma Corporation described 361 aromatic carboxamides, exemplified by compound **44** (IC_50_ = 4nM, Figure 4), with TRPM8 IC_50_ values from 0.2 to 2760 nM in their biological assays, which were used for the treatment of different types of chronic pain [84]. Dompe Farmaceutici S.p. A. related a collection of 32 4-hydroxy-2-phenyl-1,3-thiazol-5-yl methanone derivatives of general formula **45** (Figure 4), where R^1^ were (un)substituted 5–7 membered aliphatic or aromatic heterocyclic groups, containing 1–4 heteroatoms selected from S, O, and N; and R^2^ are H, alkyl, F, Cl, or OH. They claimed to have interest in these compounds for pain, neurodegeneration, ischemia, and psychiatric disorders, among other pathologies associated with TRPM8 [85]. RaQualia Pharma Inc. synthesized some imidazolinone derivatives with good pharmacokinetic properties, low toxicity, and IC_50_ values in the µM range, having the general formula **46** (R^4^ = H, alkyl, cicloalkyl or haloalkyl) (Figure 4) [86]. A member of this collection, RQ-00434739 (structure not disclosed), showed efficacy in reducing oxaliplatin-induced cold allodynia in rats and monkeys and inhibited icilin-induced wet-dog shaking in rats. Recent studies suggested that RQ-00434739 is a potent and selective antagonist of the d channel (IC_50_ = 14 nM, ˃100-TRPM8 versus TRPA1, TRPV1, TRPM2, Nav1.3, Nav1.5, Nav1.7, Cav2.2, Cav3.2), and a promising drug for bladder disorders without affecting body temperature. The intravenous administration of 1 mg/kg of RQ-00434739 blocks the hyperactivity of C-fibers induced by l-menthol and inhibits prostaglandin E2 induced hyperactivity of primary bladder afferent nerves in rats [87]. Dompe Farmaceutici S.p. A. described DFL23448, (**47**, IC_50_ = 10 nM, Figure 4) as a selective blocker of the TRPM8 channel (Figure 4) [88]. In in vivo experiments in rats, the phenyl thiazole derivative **47** (Figure 4) was able to reduce icilin-induced wet-dog shakes (10 mg/kg, iv), block bladder overactivity and prolong the storage phase of micturition [89]. Recently, Caro et al. confirmed that DFL23693 and DFL23448 (**47**, Figure 4) produce considerable antinociceptive effects in orofacial pain hypersensitivity induced by formalin and in neuropathic pain [90]. In a 2017 patent, the commercially available TRPM8 antagonist SML0893 (**48**, Figure 4), was administered alone or in combination with other substances to treat or prevent ocular pain or discomfort [91]. In addition, in a new invention, sulfonamide compounds (i.e., compound **49**, Figure 4) were used to treat or prevent vasomotor symptoms, such as hot flashes, because they lowered core body temperature [92].

It is interesting to note that a recent study assessed that the reduced proliferation and migration induced by the well-known AMTB antagonist (**50**, Figure 4) on breast cancer cell lines was due to the inhibition of NaV isoforms (NaV1.1–1.9) rather than TRPM8 [93].

**Figure 5:**
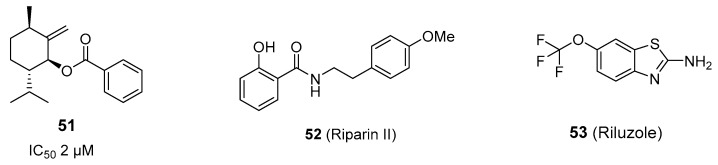
Natural product-derived and marketed drug TRPM8 antagonists.

As mentioned in the previous section, an unsaturated menthol benzoate derivative, **51** (Figure 5), related to the agonist **6** (Figure 1), showed dose-dependent inhibition of the TRPM8 currents with an IC_50_ of 2 ± 1 µM [32]. Other natural products, such as cannabigerol and some phenethyl analogue displayed nonselective antagonist properties in metabotropic CB1 and CB2 receptors and several thermo TRPs, including TRPM8 [94]. Similarly, the alkaloid riparin II (**52**, Figure 5) has important anti-inflammatory activity and produces pain relief in classical models of nociception, effects that seem mediated by a plethora of targets (i.e., TRPV1, TRPA1, TRPM8, and ASIC channels, and bradykinin and histidine receptors, among other [95].

The commercial drug Riluzole (**53**, Figure 5), used in the treatment of amyotrophic lateral sclerosis, was able to block cold allodynia alterations and mitigate mechanical allodynia produced by the antitumoral oxaliplatin, via inhibition of TRPM8 overexpression in DRG neurons (Figure 5) [96], thus constituting a new example of drug repurposing. No direct interaction with TRPM8 was described, but an indirect action by inhibiting sodium and calcium channels is suggested.

## 4. TRPM8 3D Structure

The human TRPM8 gene encodes a transmembrane protein of 1104 residues, whose functional quaternary structure is a homotetramer channel [97]. The transmembrane (TM) part is formed by six helices (S1–S6). The first four helices (S1–S4) establish the voltage sensor module and contain the binding sites for menthol and icilin [98]. The last two TM helices (S5–S6) form the pore module, which is distinguished by a highly conserved hydrophobic region and a conserved aspartate residue, responsible for ion selectivity, and whose neutralization results in a non-functional channel.

Until very recently, the knowledge of the structure–function of TRPM8 channels came from mutagenesis experiments and molecular modeling studies. The main finding from mutagenesis studies was the recognition of Y745, located in the middle of putative transmembrane segment 2, as a crucial residue for menthol binding and activation [98]. This residue was also necessary for cold and voltage activation of the TRPM8 currents, and for blockade with SKF96365, but not by other anatgonists such as BCTC, capsazepine, clotrimazole, and econazole [99]. Similar results were found in molecular simulations, where menthol and SKF96365 easily connected with the Y745 residue of the binding site, while BCTC did not bind it [100], thus suggesting the existence of allosteric points of interaction. Another study of molecular dynamic simulations also demonstrated that agonists and antagonists act in different ways. On one hand, the agonist binding triggers many conformational shifts which induce the oncoming between segments S3 and S4, ending with an extension of the S4 segment. In contrast, most antagonists occupy the binding site or block the entrance, without binding to Y745 or R802. The AMTB/TRPM8 complex is stabilized by a key charge transfer interaction with R816 plus a set of hydrophobic and π/π contact with apolar residues surrounding the binding site [101].

On the basis of the cryo-electron microscopy structure of TRPV1, several groups generated homology models of TRPM8. Thus, one of these models, along with many TRPM8 mutants, suggested that D920 residue is essential for the DDDD ring function and that the two DDDD rings could structurally participate in the stabilization of the pore through making repulsive forces [102]. Another model, assisted by the generation of chimeric TRPM8 channels containing segments of the S6-TRP box linker of TRPV1, allowed the identification of Y981 residue as important for channel function and suggested that intermonomer interactions between the TRP domain and the S4–S5 linker could influence channel gating [103].

**Figure 6:**
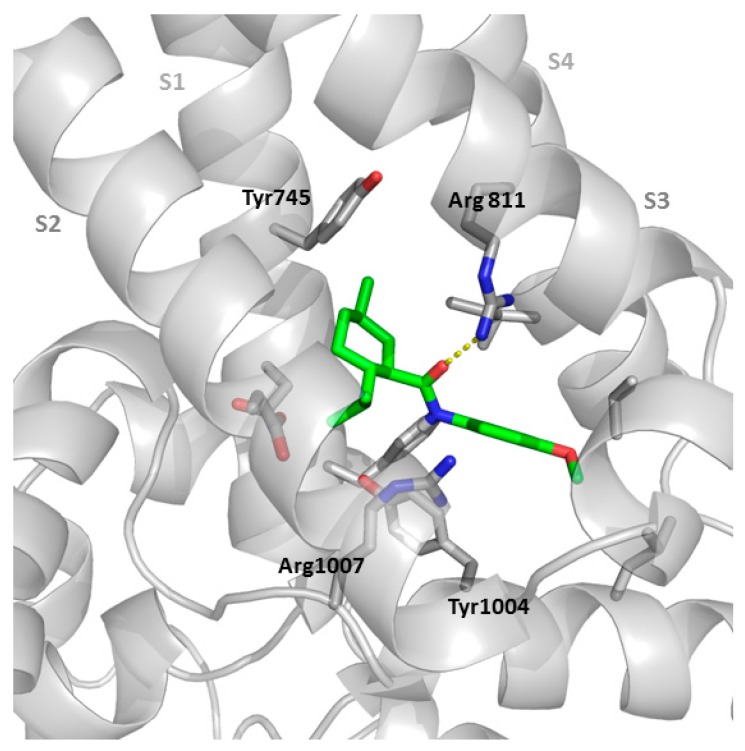
Partial structure of the TRPM8 (gray)/WS-12 (green) complex (generated from 6NR2, https://www.rcsb.org/structure/6NR2).

Quite recently, the structure of full-length TRPM8 protein from the collared flycatcher *Ficedula albicollis* (TRPM8_FA_) was solved at 4.1 Å resolution using a cryo-electron microscopy by Seok-Yong Lee et al. [104]. The protein TRPM8_FA,_ containing three mutations (F535A, Y538D, Y539D), in order to avoid proteolysis, was frozen and imaged using a transmission electron microscope [104]. The final model for TRPM8_FA_ comprised amino acids 122–1100, with multiple loops missing, and several regions (three β-strands in pre-MHR (pre-melastatin homology regions), C-terminal domain helix 1, and the C-terminal coiled coil) built as polyalanine. The TRPM8_FA_ homotetramer was shaped in a three-layered architecture, with the transmembrane channel domain (TMD) at the top, and two layers within the cytosolic domains (CD). The TMD of TRPM8_FA_ was quite similar to that of TRPV1/TRPV2, with a voltage sensor (VSLD) composed of transmembrane helical segments S1–S4, a pore domain formed by helices S5 and S6, and a pore helix [105,106]. However, some differences with other TRPs were found: (a) the pore was positioned away from the central axis and the selectivity filter organization seemed different (much longer pore loop), (b) only α-helical elements were found in the TMD (straight S4 α–helix connected to S5 through a short turn), and (c) the three short helices between S1 and the cytosolic pre-S1 helix, located at the membrane region, were not present in other TRPs. The authors also postulated that TRPM8_FA_ makes more extensive inter- and intramonomer interactions than other TRPs, and that TRPM8 voltage-sensing motion is different from canonical voltage-gated ion channels. Contrary to previous studies, the key residue Y745 was located at S1 and not at S2, but still in the voltage-sensing like domain (VSLD) cavity, and the basic interface created among TRP domain, pre-S1 helix, and N-terminal MHR4 domain was postulated as a plausible point of interaction for phosphatidylinositol (4,5)-bisphosphate (PIP_2_). Further structural studies by the same group identified the binding sites for icilin and the menthol analogue WS-12, in complexes also containing the allosteric effector PIP_2_ [107]. The reconstruction of the TRPMN8_FA_/PIP_2_/WS-12 complex (4.3 Å resolution) identified a strong density within the VSLD cavity, corresponding to the WS-12 agonist, and proposed an H-bond between the WS12 central amide and R841 side chain (S4) and other direct interactions involving Y1004 (TRPD) and R1007 (TRPD) of the protein, while Y745 (S1) was at the top of the binding site (Figure 6). A mutated protein, TRPMN8_FA-AG_ (A805G), able to be activated by icilin, was used to obtain the corresponding complex, which also required the presence of Ca^2+^ (TRPMN8_FA-AG_ /PIP_2_/icilin/ Ca^2+^). Again, the agonist was found at a cavity between VSLD and TRPD, flanked by Y745 (S1) and Y1004 (TRPD), and with side-chains of residues R841 and H844, both at S4, stablishing interactions with icilin. The interaction of H44 with icilin was not observed in the case of WS-12 and could be behind the dependence of icilin activation to intracellular pH. In the complexes, the PIP_2_ molecule was located at a highly basic region, comprising Arg and Lys residues of pre-S1 helix, MHR4, S4, S5, and the TRP domain. Although two different PIP2 binding modes were captured, it seems that in both cases there was an allosteric coupling between the PIP_2_ and the agonist. In the icilin complex, the binding of PIP_2_ by the VSLD region prompted an α- to 3_10_-helix transition in S4, and the bending and movement of S5 and TRPD, which could be responsible for the ulterior rearrangement of S6 for allowing channel gaiting.

A homology model of human TRPM8, generated from the TRPM8_FA_ structure as template, was used to performed Induced Fit Docking studies with tryptamine and tryptophan antagonists **36** and **38** (Figure 4) [76]. The simulations indicated that the indole NH of **36** (Figure 4) formed a H-bond with D781 side-chain, the α–N atom interacted with E782 residue, and a phenyl ring of the N-Bn_2_ moiety engaged cation−π interactions with R842 or R1008. In addition, some aromatic residues of the protein (Y1005, F839, and F744) established π−π interactions with the aromatic rings of compound **36** (Figure 4). The tryptophan analogue **38** (Figure 4), bound the same region and displayed similar interactions, but one of the π−π interactions was with the Y745, reported as a critical residue for the binding of menthol and SKF963635, as mentioned previously. These studies point to a competitive mechanism of action for this family of antagonists.

## 5. Conclusions and Perspectives

The knowledge on the importance of the TRPM8 channels in human physiology, its involvement in numerous pathologies, and the discovery of new chemical compounds for their modulation, continues growing day by day. Here, we have compiled recent examples of agonists and antagonists that surely will contribute in the near future, either as pharmacological tools for a better comprehension of the implications of TRPM8 in pathophysiological processes or TRPM8 as a target for the development of new drugs.

The application of rational design methodologies to the search and optimization of TRPM8 modulators seems closer. Thus, the first electron cryo-microscopy structures of TRPM8, in complex with two types of agonists [107], will undoubtedly help in the design of improved agonists, and probably will trigger future extension to complexes with different families of antagonists. These structures will certainly contribute to define probable points of interaction for known antagonists, thus fostering their modification trough structure-based, computer-assisted rational strategies. Molecular biology will also contribute with the identification of novel structural domains important for TRPM8 functions, such as the prominent role of pore loop in cool-induced responses, and therefore reinforcing the idea that the cold activation process is unique [108].

A better knowledge on the interrelation of TRPM8 and other ion channels could also assist in envisaging new approaches for the treatment of TRPM8-mediated diseases. For instance, the activation of TRPM8 channels with menthol [109], and more importantly, the coordinated activation of TRPM8 and α3β4-nAChRs could constitute attractive strategies for the treatment of obesity, type 2 diabetes, and related metabolic diseases [110].

The location, expression or morphological changes of the TRPM8 channels in different tissues, such as eyes [111], salivary glands [112], the brain [113], or the oropharyngeal system [17], among others, open new opportunities for the treatment of diseases related to these systems. Similarly, the presence and function of TRPM8 in macrophages, and the role that these channels play in T cell activation imply novel TRPM8-based opportunities for immunomodulatory intervention [114,115].

A new TRPM8 isoform, termed 4TM-TRPM8, has been identified in mitochondria-associated endoplasmic reticulum (ER) membranes (MAMs) of keratinocytes and prostate epithelial cells [116]. These channels, which have an unconventional structure with four transmembrane domains (TMs) instead of the regular six TMs, are involved in the regulation of Ca^2+^ concentrations in mitochondria and ER. To the best of our knowledge, there is neither information about selective agonists/antagonists nor three-dimensional structural information yet for these channels.

From all these recent experimental evidences, and with the help of the already discovered modulators and the new ones that will probably follow soon, we can anticipate that the field of TRPM8 channels will constantly evolve in coming years.
